# Neuroprotective effect of activated 5′-adenosine monophosphate-activated protein kinase on cone system function during retinal inflammation

**DOI:** 10.1186/s12868-016-0268-5

**Published:** 2016-06-10

**Authors:** Mamoru Kamoshita, Kaoru Fujinami, Eriko Toda, Kazuo Tsubota, Yoko Ozawa

**Affiliations:** Laboratory of Retinal Cell Biology, Department of Ophthalmology, Keio University School of Medicine, 35 Shinanomachi, Shinjuku-ku, Tokyo, 160-8582 Japan; Department of Ophthalmology, Keio University School of Medicine, 35 Shinanomachi, Shinjuku-ku, Tokyo, 160-8582 Japan

**Keywords:** Retina, Cone photoreceptor, ERG, Inflammation, AMPK, AICAR

## Abstract

**Background:**

Retinal inflammation can cause retinal neural disorders. In particular, functional disorder in the cone photoreceptor system influences visual acuity. However, the underlying mechanism is not yet fully understood. In this study, we evaluated cone system function and the role of 5′-adenosine monophosphate-activated protein kinase (AMPK) during retinal inflammation.

**Results:**

Six to eight-week-old male C57BL/6 mice received an intraperitoneal injection of lipopolysaccharide (LPS) to induce retinal inflammation, and were treated with an AMPK activator, 5-aminoimidazole-4-carboxamide ribonucleoside (AICAR; 250 mg/kg body weight) or phosphate-buffered saline as vehicle 3 h before the LPS injection. The b-wave of the photopic electroretinogram, which represents cone system function, was decreased 24 h after LPS injection and this reduction was suppressed by AICAR treatment. At this time point, there was no remarkable morphological change in the cone photoreceptor cells. At 1.5 h after LPS injection, the retina mRNA levels of an inflammatory cytokine, *Tnf*-*α*, were increased, and those of a regulator of mitochondrial biogenesis, *Pgc1*-*α*, were decreased. However, AICAR treatment suppressed these changes in mRNA expression. Immunohistochemistry showed that induction of glial fibrillary acidic protein expression was also suppressed by AICAR 24 h after LPS injection. Furthermore, the mouse cone photoreceptor-derived cell line 661W was treated with AICAR to increase the level of phosphorylated and activated AMPK. After 3 h of AICAR incubation, 661W cells showed decreased *Tnf*-*α* mRNA levels and increased *Pgc1*-*α* mRNA levels.

**Conclusion:**

AMPK activation has a neuroprotective effect on cone system function during inflammation, and the effect may, at least in part, involve the regulation of inflammatory cytokines and mitochondrial condition.

## Background

Retinal inflammation is involved in the majority of retinal diseases such as age-related macular degeneration and diabetic retinopathy, which seriously threaten vision. This is because inflammatory signaling in the retina can cause retinal neural disorders and visual disorders [1]. However, the molecular mechanism underlying retinal inflammation and its consequent effects on vision is not fully understood, thus limiting the development of an effective therapeutic approach. Here, we focus on activated 5′-adenosine monophosphate-activated protein kinase (AMPK), an essential signaling molecule for cellular energy and mitochondrial homeostasis, which decreases in the retina during inflammation [2]. We previously demonstrated that the level of phosphorylated and activated AMPK was reduced and an inflammatory transcription factor, NF-κB, was activated in the retina which indicates the retinal inflammation [2], and rod system function is impaired in the endotoxin-induced uveitis (EIU) mouse model induced by lipopolysaccharide (LPS) [2–5]. In this model, major blood vessels are dilated and monocytes adhere to them in the retina, which also indicates retinal inflammation [6–9]. However, whether cone system dysfunction is induced during inflammation has yet to be seen. In this study, we analyzed the photopic electroretinogram (ERG), which represents cone system function, during inflammation in the EIU mouse model, and evaluated the ability of AMPK to preserve cone system function.

## Methods

### Animals

Six to eight-week-old male C57BL/6 mice (CLEA Japan, Tokyo, Japan) were maintained in an air-conditioned room under a 12-h dark/light cycle, with free access to food and water. The mice were randomly divided into three groups; control non-EIU with vehicle treatment, EIU with vehicle treatment, and EIU with AICAR treatment groups. The mice received a single intraperitoneal injection of 6.0 mg/kg body weight (BW) LPS from *Escherichia coli* (Sigma-Aldrich; St. Louis, MO, USA) in phosphate-buffered saline (PBS) to generate an EIU model which develops retinitis as well as uveitis [2–9]. The mice were treated with the specific AMPK activator 5-aminoimidazole-4-carboxamide ribonucleoside (AICAR; Santa Cruz Biotechnology; Santa Cruz, CA, USA) at 250 mg/kg BW or PBS as vehicle 3 h before the LPS injection. Reduction in the activated AMPK level during inflammation and its suppression by AICAR were confirmed previously [2]. All animal experiments were conducted in accordance with the Association for Research in Vision and Ophthalmology (ARVO) Statement for the Use of Animals in Ophthalmic and Vision Research and the protocol for this study was approved by the Keio University Institutional Animal Care and Use Committee (permission no. 08002) (Tokyo, Japan). For accuracy, all the experiments were repeated 2 or 3 times, but the number of the animals used in each experiment was minimized to follow the guideline. There were no important adverse events during the experiments.

### Electroretinogram

The mice were anesthetized with 60 mg/kg BW of pentobarbital sodium (Dainippon Sumitomo Pharmaceutical Co., Tokyo, Japan) and kept on a heating pad throughout the experiment. Photopic ERGs were recorded with contact lens electrodes placed on the cornea (Mayo; Inazawa, Japan) after 10 min of light adaptation. Flash stimuli ranging from 0.46 to 2.89 log cds m^−2^ were used for recording with a background of 30 cds m^−2^ (PowerLab System 2/25; AD Instruments; New South Wales, Australia), and the results of 20 single-flash trace trials were averaged. Eight mice for each group were used.

### Real time RT-PCR

Total RNA was isolated from the retina or the cell line (see below) with TRIzol reagent (Invitrogen, Carlsbad, CA, USA) and was reverse-transcribed using the High Capacity RNA-to-cDNA Master Mix (Applied Biosystems; Foster City, CA, USA), according to the manufacturer’s instructions. Real-time polymerase chain reaction (PCR) was performed using the StepOnePlus™ Real-Time PCR system (Applied Biosystems), and the expression of the gene encoding the inflammatory cytokine tumor necrosis factor-alpha (*Tnf*-*α*) was quantified using the delta–delta cycle threshold method, and normalized to *Gapdh* expression. The forward and reverse primer sequences for *Tnf*-*α* were 5′-GCCACCACGCTCTTCTGTCTA-3′ and 5′-GATGAGAGGGAGGCCATTTG-3′, those for peroxisome proliferator-activated receptor gamma coactivator 1-α (*Pgc1*-*α*) were 5′-GATGAATACCGCAAAGAGCA-3′ and 5′-AGATTTACGGTGCATTCCTCA-3′, and those for *Gapdh* were 5′-AACTTCGGCCCCATCTTCA-3′ and 5′-GATGACCCTTTTGGCTCCAC-3′. Six mice for each group were used to prepare the retinal samples, and 6 samples of 661W cells in each group were analyzed.

### Histochemistry

Retinal sections (10-μm thick) were fixed in 4 % paraformaldehyde, and incubated with rhodamine-conjugated peanut agglutinin (Vector Laboratories; Burlingame, CA, USA) at room temperature for 1 h. Alternatively, they were incubated with rabbit anti-GFAP antibody (1:500; DAKO, Carpinteria, CA, USA) overnight at 4 °C, followed by incubation with Alexa 488-conjugated goat anti-rabbit IgG at room temperature for 1 h. All the sections were examined under a microscope equipped with a digital camera (Leica Microsystems, Wetzlar, Germany). Four mice for each group were used to prepare the retinal sections.

### Culture

The 661W cells (a kind gift from Dr. Muayyad R. Al-Ubaidi, University of Oklahoma Health Sciences Center, Oklahoma City, OK, USA) were maintained in Dulbecco’s modified Eagle’s medium (DMEM; Sigma-Aldrich, St. Louis, MO, USA) supplemented with 10 % fetal bovine serum, penicillin (100 units/ml), and streptomycin (100 μg/ml) at 37 °C under a humidified atmosphere of 5 % CO_2_. The cells were incubated with either AICAR (0.5 or 1 mM) or vehicle in serum-free DMEM for 3 h. Six samples for each group were analyzed.

### Immunoblot analyses

Samples from the retina or the cell line were isolated and placed in lysis buffer, including protease inhibitor cocktail (Complete, EDTA-free; Roche, Mannheim, Germany) and phosphatase inhibitor cocktails 2 and 3 (Sigma-Aldrich) to prepare the lysate, and immunoblot analyses were performed as described elsewhere [2]. Briefly, the proteins were separated by electrophoresis and electrically transferred to a polyvinylidene fluoride membrane (Immobilon-P; Millipore; Bedford, MA, USA). The membrane was blocked with 5 % skim milk in Tris-buffered saline with Tween or TNB blocking buffer [0.1 M Tris–HCl, pH 7.5, 0.15 M NaCl, 0.5 % TSA Blocking Reagent (PerkinElmer Life Sciences; Waltham, MA, USA)], and then incubated overnight at 4 °C with a rabbit anti-phospho-AMPKα (1:1000; Cell Signaling Technology) or rabbit anti-AMPKα (1:200; Cell Signaling Technology) for normalization. Six samples for each group were analyzed.

### Statistical analyses

All results are expressed as the mean ± SD. One-way ANOVA with Tukey’s post hoc test was used to assess the statistical significance of differences between the groups. P values <0.05 were considered as significant.

## Results

### Impairment of cone system function during inflammation and its suppression by AICAR

**Fig. 1:**
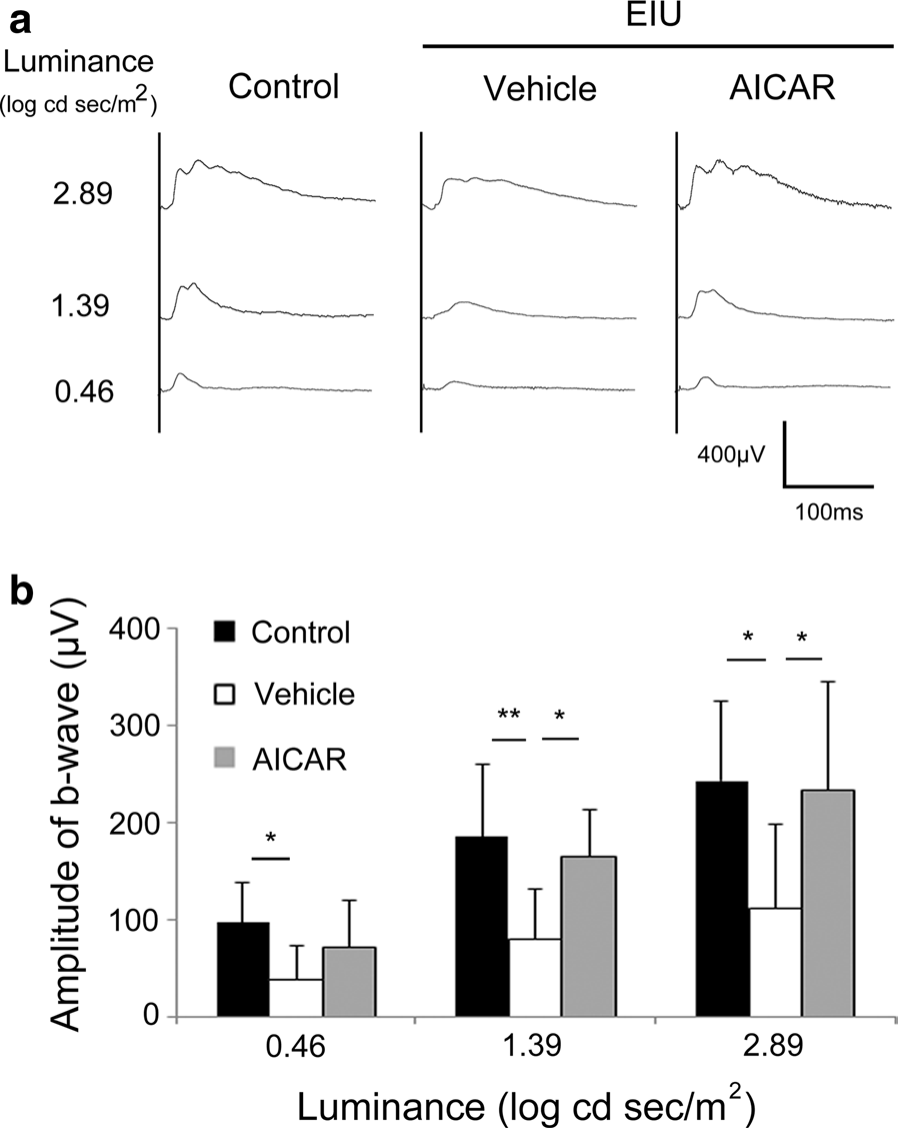
Cone responses recorded by photopic electroretinogram during inflammation. Photopic electroretinogram (ERG) data 24 h after lipopolysaccharide (LPS) injection. **a** Representative photopic ERG waveforms from an individual mouse. **b** The b-wave amplitude showed significant reduction, which was prevented by 5-aminoimidazole-4-carboxamide ribonucleoside (AICAR) treatment. All groups, n = 8. All results are expressed as the mean ± SD. One-way ANOVA with Tukey’s post hoc test was used to assess the statistical significance of differences. P < 0.05 was regarded as significant. *P < 0.05. **P < 0.01

In these mice, we first evaluated the photopic ERG 24 h after LPS injection when rod photoreceptor responses in ERG had already been clearly reduced in this model [2]; Representative waveforms from an individual mouse in each group and means of each group are shown in Fig. 1a, b, respectively. The photopic ERG b-wave, which represents cone system function [10, 11], was decreased in the EIU model, and this reduction was suppressed by AICAR treatment (b-wave amplitudes in non-EIU with vehicle treatment, EIU with vehicle treatment, and EIU with AICAR treatment groups: 97.5 ± 41.2, 39.3 ± 34.7, and 71.6 ± 48.8 μV in response to 0.46 log cds m^2^ light stimuli; 185.5 ± 75.6, 80.5 ± 51.1, and 165.5 ± 48.0 μV in response to 1.39 log cds m^2^ stimuli; 243.0 ± 81.9, 112.1 ± 86.6, and 233.7 ± 110.9 μV in response to 2.89 log cds m^2^ stimuli) (Fig. 1a, b). Under this condition, rod photoreceptor cells are saturated and cannot be detected with light adaptation. These findings indicate that cone system dysfunction occurred during inflammation via reduced AMPK activation. Although the outer segment (OS) length is shortened in the rods during inflammation [2], the OS length of the cones was maintained in the current study. This was measured in the cryosections obtained 24 h after LPS injection that showed a positive reaction with the cone marker peanut agglutinin (data not shown). Therefore, cone system dysfunction was observed in this inflammatory model, even without remarkable morphological changes.

### Anti-inflammatory effect of AICAR in the retina

**Fig. 2:**
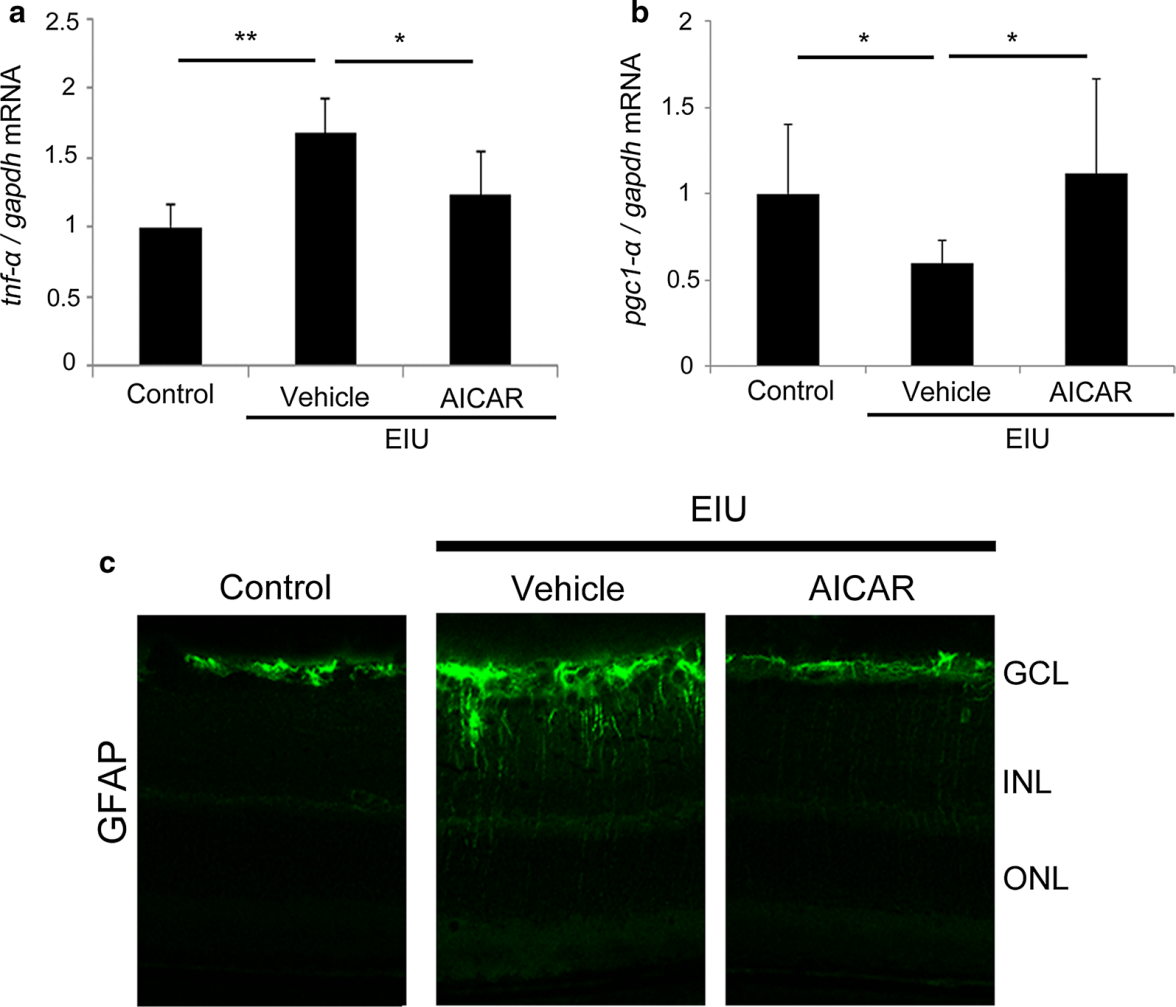
Anti-inflammatory effect of AICAR in the retina. **a**, **b** The retinal samples were obtained 1.5 h after lipopolysaccharide (LPS) injection and subjected to real-time polymerase chain reaction (PCR). **a** The mRNA level of *Tnf*-*α* was increased in the retina, and the increase was significantly suppressed by 5-aminoimidazole-4-carboxamide ribonucleoside (AICAR) treatment. **b** The mRNA level of *Pgc1*-*α* in the retina was decreased in the endotoxin-induced uveitis (EIU) model, and this decrease was attenuated by AICAR. **c** Immunohistochemistry showing that the enhanced expression of glial fibrillary acidic protein (GFAP) in the retina 24 h after LPS injection was suppressed by AICAR treatment. **a**–**c** All groups, n = 6. **a**, **b** All results are expressed as the mean ± SD. One-way ANOVA with Tukey’s post hoc test was used to assess the statistical significance of differences. P < 0.05 was regarded as significant. *P < 0.05. **P < 0.01

Next, we analyzed the inflammatory signaling in the retina of the model mice with or without AICAR treatment. For this purpose, total RNA was isolated from the retina 1.5 h after LPS injection; this is the time point when the inflammatory transcription factor, NF-κB, was activated in the retina in response to the reduction of activated AMPK, and this change was suppressed by AICAR, as we have previously shown [2]. The mRNA of *Tnf*-*α* was upregulated in the retina during inflammation (*P* < 0.01), and was suppressed by AICAR treatment (*P* < 0.05) (Fig. 2a). Furthermore, mRNA expression levels were evaluated for a regulator of mitochondrial biogenesis, *Pgc1*-*α*, in the same retinal samples using real-time PCR. *Pgc1*-*α* mRNA levels decreased during inflammation (*P* < 0.05), and were preserved by AICAR treatment (*P* < 0.05) (Fig. 2b).

We also analyzed expression of GFAP, a marker for reactive glia during retinal inflammation, by immunohistochemistry (Fig. 2c). We found that GFAP expression induced in the retina during inflammation was suppressed by AICAR, suggesting that AICAR suppresses the inflammatory glial reaction in the retina.

### Effect of AICAR on *Tnf*-*α* and *Pgc1*-*α* mRNAs in the photoreceptor cell line

**Fig. 3:**
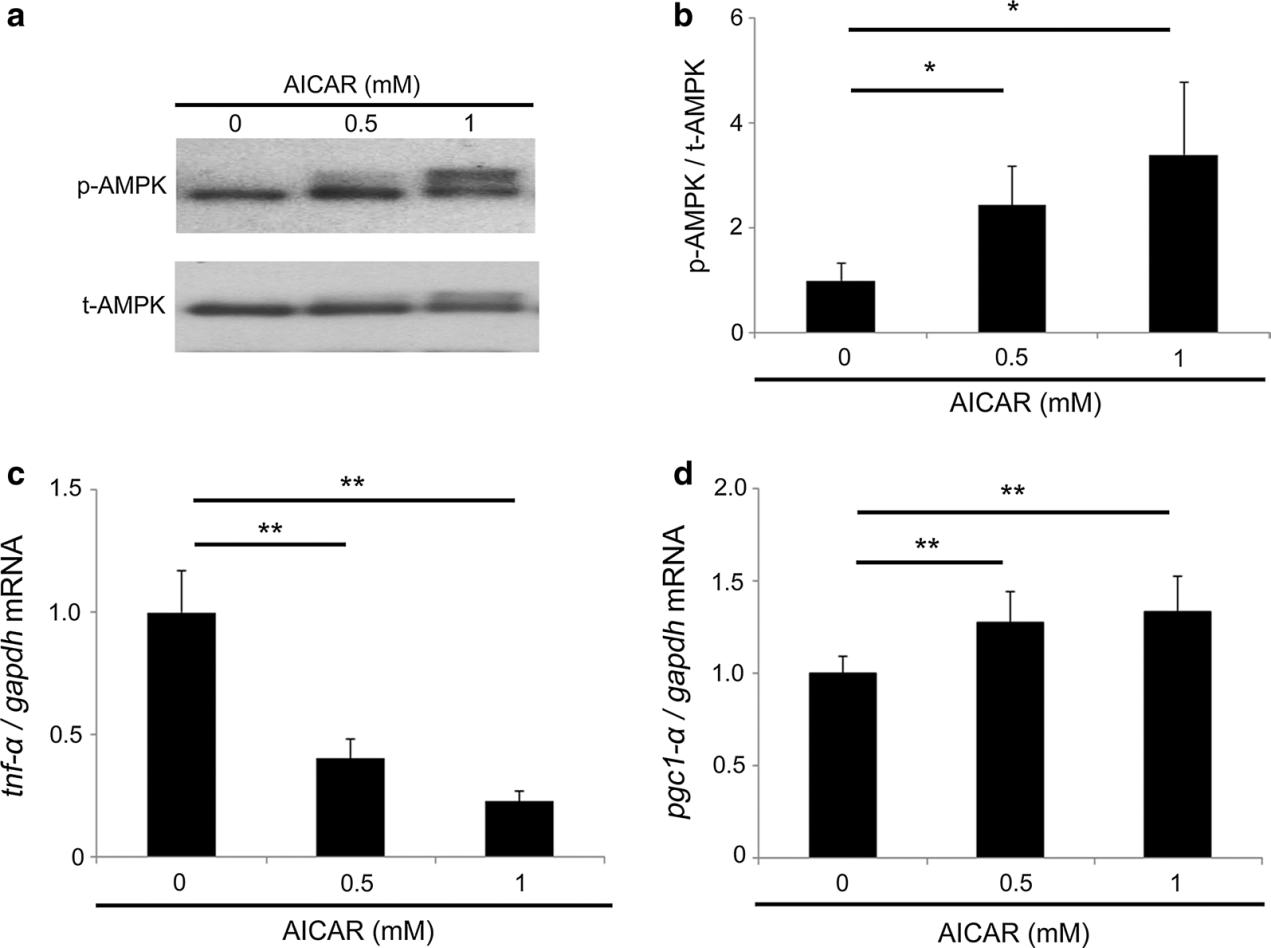
The effect of AICAR on *Tnf*-*α* and *Pgc1*-*α* expression. **a**–**d** The samples of 661W cells were obtained 3 h after incubation with AICAR. **a**, **b** Immunoblot analyses. The level of phospho-5′-adenosine monophosphate-activated protein kinase (AMPK) was upregulated with AICAR treatment at concentrations of 0.5 mM and 1 mM. **c**, **d** Real-time PCR. The mRNA level of *Tnf*-*α* was decreased (**c**) and that of *Pgc1*-*α* was increased (**d**) by AICAR treatment. All groups, n = 6. All results are expressed as the mean ± SD. One-way ANOVA with Tukey’s post hoc test was used to assess the statistical significance of differences. P < 0.05 was regarded as significant. *P < 0.05. **P < 0.01

To elucidate the role of AICAR in the cone photoreceptor cells, we performed in vitro experiments using the mouse cone photoreceptor-derived cell line 661W. The levels of phosphorylated and activated AMPK in the cells incubated with either AICAR (0.5 mM or 1 mM) or vehicle in serum-free DMEM for 3 h were confirmed by immunoblot analyses. The level of activated AMPK was increased by AICAR treatment (*P* < 0.05) (Fig. 3a, b). At the same time point, *Tnf*-*α* and *Pgc1*-*α* mRNA levels were measured by real-time PCR; *Tnf*-*α* levels decreased (*P* < 0.01) (Fig. 3c) and *Pgc1*-*α* levels increased (*P* < 0.01) (Fig. 3d) from the AICAR treatment.

## Discussion

We demonstrated that cone system function was decreased after LPS injection. This reduction was suppressed by AICAR treatment, although there was no remarkable morphological change in the cone photoreceptor cells. The retina mRNA levels of an inflammatory cytokine, *Tnf*-*α*, were increased, and those of a regulator of mitochondrial biogenesis, *Pgc1*-*α,* were decreased at the earlier time point after LPS injection. Immunohistochemistry showed that induction of GFAP expression was also suppressed by the time point when cone system dysfunction was recorded. However, AICAR treatment suppressed all these changes in the EIU mice. Furthermore, in the mouse cone photoreceptor-derived cell line 661W, induction of activated AMPK by AICAR decreased *Tnf*-*α* mRNA levels and increased *Pgc1*-*α* mRNA levels.

Given that TNF-α-induced neurotoxicity can be mediated by rapid mitochondrial dysfunction [12], the cone system dysfunction during inflammation may be related to this pathway; in fact, in this study, *Tnf*-*α* was induced and *Pgc1*-*α* was repressed during the inflammation that caused cone system dysfunction. Moreover, AMPK activation played a direct role in suppressing these effects, suggesting a critical role of activated AMPK. The mechanism suggested by these results is as follows: reduction in activated AMPK may induce an inflammatory cytokine, TNF-α, during inflammation, which would disrupt mitochondrial homeostasis, at least in part, in the cone photoreceptor cells, thereby impairing cone system function during retinal inflammation. AMPK activation may be an intrinsic self-defense mechanism for cone photoreceptor cells to maintain a healthy condition, and any breakdown of this mechanism during inflammation would lead to dysfunction.

Furthermore, the fact that GFAP expression induced during inflammation was also suppressed by AICAR implicates an indirect role for Müller glial cells in the neuroprotective effect on the cone photoreceptor neurons during inflammation. TNF-α can be expressed by both photoreceptors and Müller glial cells [13]. Nonetheless, the present in vitro results suggest a direct effect of AMPK activation, at least in part, in the protection of cone photoreceptor neurons.

Functional impairment in our model could have preceded any visible morphological damage, although minimum architectural changes cannot be excluded. The discrepancy between function and morphology is not uncommon in human subjects with uveitis [14]. Indeed, Holder et al. [14] reported that the cone functional damage preceded morphological changes in patients with inflammatory disorders, and, in some cases, improvement following treatment preceded visible clinical signs of recovery. This indicates that an inflammatory reaction can cause cone system dysfunction, which may cause central visual impairment in humans even with no marked architectural alteration. Thus, cone system dysfunction could be an early sign of inflammatory disease, as well as a sensitive parameter for measuring treatment efficacy, although the findings in the current study were obtained from mice and not human, thus further study is required to fully discuss the human findings.

## Conclusion

Cone system dysfunction may occur during inflammation, at least in part, through decreased levels of activated AMPK. An AMPK activator, AICAR, protected the function of the cone photoreceptor system during inflammation, repressing an inflammatory cytokine, *Tnf*-*α*, and preserving a regulator of mitochondrial biogenesis, *Pgc1*-*α*. These results suggest that preserving activated AMPK levels in the retina may be a new therapeutic approach for protecting cone system function during inflammation.

## Abbreviations

AICAR: 5-aminoimidazole-4-carboxamide ribonucleoside
AMPK: 5′-adenosine monophosphate-activated protein kinase
EIU: endotoxin-induced uveitis
ERG: electroretinogram
LPS: lipopolysaccharide
OS: outer segment
PBS: phosphate-buffered saline

## References

[1] Ozawa Y, Kurihara T, Tsubota K, Okano H (2010). Regulation of posttranscriptional modification as a possible therapeutic approach for retinal neuroprotection. J Ophthalmol.

[2] Kamoshita M, Ozawa Y, Kubota S, Miyake S, Tsuda C, Nagai N (2014). AMPK-NF-κB axis in the photoreceptor disorder during retinal inflammation. PLoS ONE.

[3] Kurihara T, Ozawa Y, Shinoda K, Nagai N, Inoue M, Oike Y (2006). Neuroprotective effects of angiotensin II type 1 receptor (AT1R) blocker, telmisartan, via modulating AT1R and AT2R signaling in retinal inflammation. Invest Ophthalmol Vis Sci.

[4] Ozawa Y, Nakao K, Kurihara T, Shimazaki T, Shimmura S, Ishida S (2008). Roles of STAT3/SOCS3 pathway in regulating the visual function and ubiquitin-proteasome-dependent degradation of rhodopsin during retinal inflammation. J Biol Chem.

[5] Sasaki M, Ozawa Y, Kurihara T, Noda K, Imamura Y, Kobayashi S (2009). Neuroprotective effect of an antioxidant, lutein, during retinal inflammation. Invest Ophthalmol Vis Sci.

[6] Yang P, de Vos AF, Kijlstra A (1996). Macrophages in the retina of normal Lewis rats and their dynamics after injection of lipopolysaccharide. Invest Ophthalmol Vis Sci.

[7] Miyamoto K, Ogura Y, Hamada M, Nishiwaki H, Hiroshiba N, Honda Y (1996). In vivo quantification of leukocyte behavior in the retina during endotoxin-induced uveitis. Invest Ophthalmol Vis Sci.

[8] Nagai N, Oike Y, Noda K, Urano T, Kubota Y, Ozawa Y (2005). Suppression of ocular inflammation in endotoxin-induced uveitis by blocking the angiotensin II type 1 receptor. Invest Ophthalmol Vis Sci.

[9] Satofuka S, Ichihara A, Nagai N, Yamashiro K, Koto T, Shinoda H (2006). Suppression of ocular inflammation in endotoxin-induced uveitis by inhibiting nonproteolytic activation of prorenin. Invest Ophthalmol Vis Sci.

[10] Lei B (2012). Rod-driven OFF pathway responses in the distal retina: dark-adapted flicker electroretinogram in mouse. PLoS ONE.

[11] Lei B, Yao G, Zhang K, Hofeldt KJ, Chang B (2006). Study of rod- and cone-driven oscillatory potentials in mice. Invest Ophthalmol Vis Sci.

[12] Doll DN, Rellick SL, Barr TL, Ren X, Simpkins JW (2015). Rapid mitochondrial dysfunction mediates TNF-alpha-induced neurotoxicity. J Neurochem.

[13] Nelson CM, Ackerman KM, O’Hayer P, Bailey TJ, Gorsuch RA, Hyde DR (2013). Tumor necrosis factor-alpha is produced by dying retinal neurons and is required for Muller glia proliferation during zebrafish retinal regeneration. J Neurosci.

[14] Holder GE, Robson AG, Pavesio C, Graham EM (2005). Electrophysiological characterisation and monitoring in the management of birdshot chorioretinopathy. Br J Ophthalmol.

